# Eucaine—A Note on the New Local Anesthetic

**Published:** 1896-10

**Authors:** J. C. Clemesha

**Affiliations:** Buffalo Eye and Ear Infirmary, Buffalo, N. Y. 382 West Ferry Street


### EUCAINE—A NOTE ON THE NEW LOCAL ANESTHETIC.

By J. C. CLEMESHA, M. D., M. R. C. S., Eng., L. R. C. P., Lond.
Buffalo Eye and Ear Infirmary, Buffalo, N. Y.

IN A PAPER read at the session of the Hufeland Society, Berlin, April 16, 1896, Dr. Vinci, of Messina, described the chemical composition and properties of a new compound, which, for convenience sake, has been called eucaine.

This new drug, it is stated, is not derived from the vegetable kingdom, but is a laboratory product, and its proper name is methyl-benzoyl-tetramethyl-gamina-oxypiperidine-carbonicacid-methyal-ester, while the salt in use is the muriate which crystallises from a watery solution in permanent shiny scales, and is soluble in water to the extent of 6 per cent. Furthermore, it can be prepared in any quantity, and I believe the intention is to put it upon the market at a cost considerably below that of cocaine.

Dr. Vinci, in his communication, described eucaine as possessing the properties of cocaine as a local anesthetic, but as being less toxic and as having no effect upon the pupil.

Having read an extract of the above paper, and realising, if the statements were accurate, that an important addition had been made to the list of drugs used in ophthalmic practice, Dr. Howe procured a supply from the New York agents, and a 5 per cent. watery solution was prepared. The first day it was used at the Buffalo Eye and Ear Infirmary was in a 5 per cent. solution and in the following cases: (1) Removal of a foreign body embedded in the cornea; (2) removal of granulation after a strabismus operation; (3) the operation of tatooing the cornea.

In the foregoing cases the patients complained that the instillation was followed by a smarting, pricking sensation, lasting some minutes, while it was noted that some hyperemia of the conjunctiva was produced, which remained for half an hour or so after the anesthesia passed away. Anesthesia was complete in from five to ten minutes, none of the patients complaining of the least pain during operative manipulation. It was also noted that the pupil did not dilate, but reacted well to light, and that the corneal epithelium showed no tendency to become sodden, as it does after the application of cocaine.

The day following, Dr. Howe used it with complete success in a strabismus operation, and it has been used for extractions and iridectomies, the patients complaining of no pain whatsoever.

Later, a 1 to 2 per cent. solution was prepared, and it was found that anesthesia was as readily produced without the pricking sensation which follows the application of the stronger solutions, and these weaker solutions are being employed at the Buffalo Eye and Ear Infirmary for the removal of foreign bodies in the eye and other minor operations.

Comparison was made with a 2 per cent. solution of cocaine and a solution of eucaine of the same strength, with the result that the two drugs were of the same value as regards rapidity, duration and intensity of the anesthesia.

Eucaine has been proved to be less poisonous than cocaine. Animals treated with eucaine survived, while animals injected with the same doses of cocaine perished. The heart is in no way influenced by it, while it is a well-known fact that dangerous symptoms often arise from the use of cocaine. Professor Charteris, of Glasgow, has made comparative experiments with the hydrochlorate of eucaine and the hydrochlorate of cocaine. Watery solutions of these salts were injected into guinea-pigs, beginning with a small dose and gradually increasing it until the lethal dose was accurately ascertained. It was found, after many experiments, that the toxic dose of eucaine per kilo body weight was 0.09 gramme, and that the toxic dose of cocaine per kilo body weight was 0.068 gramme. It was also noticed that the physiological action produced by a given dose of eucaine did not follow nearly so rapidly as that which followed a similar dose of cocaine, hence it was concluded that the action of eucaine was slower in onset and less in intensity. Finally, eucaine solutions are stable and permanent and do not decompose, like those of cocaine when kept for a time.

Tropacocaine, another local anesthetic, seems to be midway between cocaine and eucaine. It is less toxic than cocaine, and on instillation produces slight smarting and some hyperemia of the conjunctiva. Mydriasis is usually absent and the anesthesia is more quickly complete and does not last as long as cocaine. Unfortunately its manufacture has been abandoned on account of the difficulty of its preparation.

Eucaine has also been employed in dentistry for the extraction of teeth ; 3 to 5 minims of a 10 per cent. solution, injected into each root to be extracted, is sufficient. It has also been used in laryngology and, in the form of an ointment, in painful skin affections.

Berger (*Revue de Therapeutique*, June 15, 1896,) thus sums up the properties of eucaine: He has employed it clinically and thinks it a useful substitute for cocaine. It is little soluble in water, but the hydrochlorate can be dissolved in water to the extent of 6 per cent. It is not so toxic as cocaine, while its anesthetic effect is fully equal to that of the latter; but, whereas cocaine produces local anemia, eucaine produces local hyperemia.

Applied to the cornea, it does not tend to produce desquamation of the superficial epithelium, which cocaine does, and it has the further advantage of not affecting the pupil or the accommodation.

A 1 per cent. solution applied to the eye causes no pain; a 2 per cent. solution causes some pricking.

Anesthesia occurs in a few minutes. The hyperemia of the conjunctiva lasts for half an hour after anesthesia passes off.

The solutions of eucaine are very stable, and so can be easily sterilised.

382 West Ferry Street.

